# GEROSCIENCE BLOOD-BASED BIOMARKER INDICES AND PHYSICAL PERFORMANCE IN OLDER ADULTS

**DOI:** 10.1093/geroni/igad104.3371

**Published:** 2023-12-21

**Authors:** Denise Houston, Deeya Pathak, Rebecca Neiberg, Jamie Justice, Barb Nicklas, Michael Miller

**Affiliations:** Wake Forest University School of Medicine, Winston-Salem, North Carolina, United States; Wake Forest University School of Medicine, Winston-Salem, North Carolina, United States; Wake Forest University School of Medicine, Winston-Salem, North Carolina, United States; XPRIZE Foundation, Winston-Salem, North Carolina, United States; Wake Forest University School of Medicine, Winston-Salem, North Carolina, United States; Wake Forest University School of Medicine, Winston-Salem, North Carolina, United States

## Abstract

Dysregulation in cellular and molecular aging biomarkers are hypothesized to contribute to worse physical performance. We examined associations between blood-based geroscience biomarker indices and physical performance in 414 older adults (77.4±4.4 years; 69% female). Physical performance measures included the expanded SPPB, 4m and 400m walk, and grip strength. Serum IL-6, CRP, TNFr1, GDF15, and insulin were measured and three biomarker indices created: 1) a quintile index calculated by summing quintile scores for each of the biomarkers; 2) a composite z-score calculated using the standardized mean of individual biomarker z-scores; and 3) a principal component analysis (PCA) to identify biomarker factor(s). The quintile index and composite z-score were highly correlated (r=0.94, p< 0.0001). PCA identified two factors: TNFr1 and GDF-15 weighted heavily on Factor 1 and IL6 and CRP weighted heavily on Factor 2. The quintile index and composite z-score were correlated with PCA Factors 1 and 2 (r>0.61, p< 0.0001). Similar standardized beta coefficients were observed for the quintile index, composite z-score, and PCA Factor 1 with 4m and 400m gait speed (all p< 0.0001); every 1 SD higher biomarker index score was associated with ~0.25 SD slower gait speed. For expanded SPPB, higher standardized beta coefficients were observed for the quintile index and composite z-score (~-0.32) than PCA Factors 1 and 2 (~-0.24; all p< 0.0001). Weak associations between all indices and grip strength were observed (p≥0.05). Geroscience biomarker indices that capture inflammation and poor stress response appear to have similar associations with worse physical performance in older adults.

